# Navigating Airway Management in Post-tracheostomy Patients: A Case Report and Review of the Literature

**DOI:** 10.7759/cureus.92996

**Published:** 2025-09-23

**Authors:** Basma Andrabi, Syeda Mariam Zehra Naqvi, Saad Ur Rehman

**Affiliations:** 1 Anesthesiology, Shaukat Khanum Memorial Cancer Hospital and Research Centre, Lahore, PAK

**Keywords:** bronchial blocker, difficult airway, double lumen tube, one-lung ventilation, post laryngectomy, tracheostomy

## Abstract

One-lung ventilation (OLV) is an essential technique in thoracic anesthesia to isolate lungs and provide optimum operating conditions. Different techniques have been described in the literature to achieve adequate surgical exposure. However, in anatomically altered upper airways, such as in post-laryngectomy patients, airway management remains challenging. Here, we report a case where OLV was successfully accomplished utilizing a double-lumen tube following a total laryngectomy and a comprehensive literature review of achieving OLV in patients with tracheostomy/laryngectomy. Informed consent was obtained from the patient, permitting the publication of this case report along with all associated clinical images and data.

## Introduction

One-lung ventilation (OLV) is a cornerstone of thoracic surgery, enabling effective lung isolation and optimizing surgical conditions for procedures such as pneumonectomy, lobectomy, video-assisted thoracoscopic surgery (VATS), esophagectomy, and thoracic aortic surgery [1]. Standard methods of achieving OLV include double-lumen tubes (DLTs), specially designed short DLTs, single-lumen tubes (SLTs), and bronchial blockers (BBs) [2]. However, achieving OLV in post-laryngectomy patients can be troublesome due to the risk of malpositioning and critical airway compromise.

An important factor to consider during device selection is the stage of the stoma, as an early stoma carries a higher risk of airway loss due to potential stoma instability. Conversely, well-matured stomas in long-term tracheostomy patients provide a comparatively stable conduit for device insertion [1,3]. The anatomy, pathology, and intended surgical technique of the patient should all be taken into consideration when selecting a device [2]. The anatomical alterations inherent in post-laryngectomy patients, including a truncated airway and the inability to perform standard orotracheal intubation, necessitate highly specialized and adaptive strategies. During the navigation of airway instrumentation in patients with post-surgical anatomy, fiberoptic bronchoscopy plays a pivotal role [4].

The application of DLTs in tracheostomized patients is fraught with limitations due to their considerable external diameter, structural rigidity, and the constrained dimensions of tracheostomy stomas, all of which amplify the risk of airway trauma [1,5].

Airway management in the context of tracheostomy and tracheostoma demands meticulous planning, given the substantial risks of airway injury, loss of ventilation, or failure to achieve adequate oxygenation [1,3-5]. In these considerations, the first priority is securing the airway, whereas lung isolation remains an independent and secondary consideration [4,5]. In cases where DLT placement would be impracticable or contraindicated, initial intubation with an SLT, followed by conversion to a DLT, if achievable, may be a reasonable compromise [5].

Herein, we report a case where OLV was successfully accomplished utilizing a DLT following a total laryngectomy. Informed consent was obtained from the patient, permitting the publication of this case report along with all associated clinical images and data.

## Case presentation

**Figure 1:**
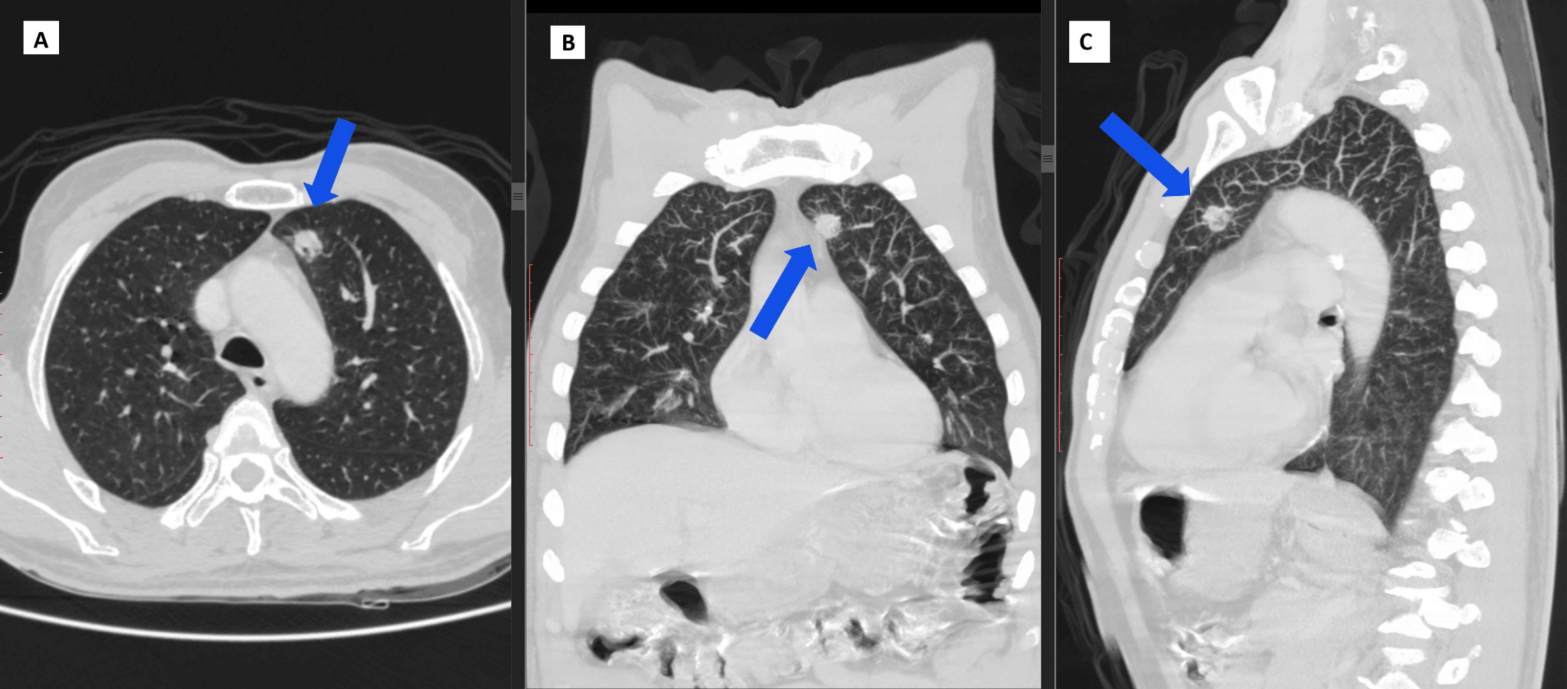
Contrast-enhanced CT chest showing a left upper lobe pulmonary mass (blue arrows) in axial (A), coronal (B), and sagittal (C) reconstructions. CT: computed tomography

A 56-year-old man who was 169 cm tall and weighed 70 kg had a history of laryngeal squamous cell carcinoma. He had a complete laryngectomy in 2021, which was followed by the formation of a permanent tracheostoma and subsequent adjuvant radiotherapy. The patient presented again with complaints of chronic cough and dyspnea for two months. Cavitary soft tissue nodules, approximately 14 × 12 mm, were found anteriorly in the left upper lobe on computed tomography (Figure 1). Following careful consideration by the multidisciplinary team, a surgical strategy was formulated involving either a left VATS or thoracotomy, with a proposed left lower lobe wedge resection or left lobectomy, necessitating OLV. Echocardiography and preoperative lab tests were unremarkable.

The patient was scheduled electively for left VATS + resection of pulmonary nodule with or without lobectomy. After careful preoperative anesthesia assessment and obtaining informed consent with explaining all risks and benefits, the patient was received in the OR, and ASA standard monitoring was applied. A 22-gauge peripheral IV cannula at the left forearm was flushed and checked with 10 ml of normal saline. Intravenous Ringer's lactate infusion was started. The patient was positioned and draped for awake thoracic epidural. Under all aseptic measures, thoracic epidural was inserted at the T5-6 level in the sitting position. Loss of resistance (LOR) was achieved at 7 cm, and the catheter length left in epidural space was 5 cm. The catheter was adequately secured.

**Figure 2:**
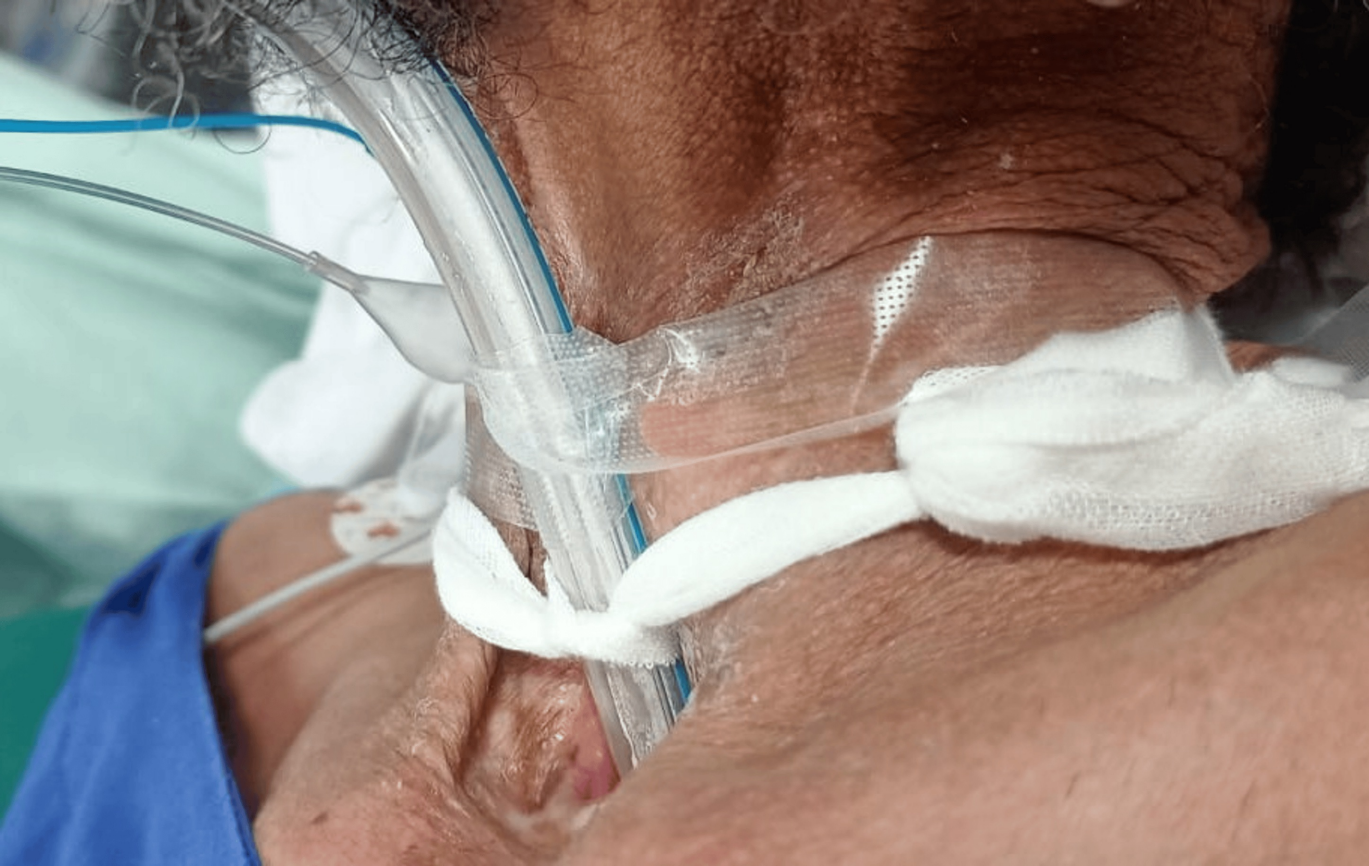
Double-lumen tube via tracheostoma

The patient was then turned supine for induction of anesthesia and preoxygenated for three minutes with five liters of oxygen via a tracheostomy mask. Following that, 2 mg midazolam, 100 mg propofol and 100 micrograms fentanyl were given intravenously. As the patient lost consciousness while maintaining spontaneous respiration, a size 7 single-lumen endotracheal tube (PVC) was inserted through the tracheostoma and inflated with 5 ml of air to avoid leak. Adequate placement was confirmed with a capnograph. The patient was then paralyzed with 50 mg atracurium intravenously. Check bronchoscopy was done to have an idea of anatomy. A pediatric bougie was then passed through the endotracheal tube, which was subsequently removed, and a left-sided DLT size 37 Fr was railroaded over the bougie (Figure 2). Placement was confirmed after tracheal cuff inflation via bilateral auscultation, capnograph, and fiberoptic bronchoscopy. After confirmation of adequate tube placement, OLV was successfully achieved by clamping the tracheal lumen.

The patient remained hemodynamically stable, and saturations were maintained throughout. FiO2 was kept at 100% initially and then gradually tapered off to maintain SpO2 more than 96%. Meanwhile, the right radial artery was cannulated for invasive beat-to-beat blood pressure monitoring, and two wide-bore intravenous cannulas were inserted in the arms. After that, the patient was turned in the lateral decubitus position and OLV was continued. Epidural was loaded with 0.125% bupivacaine 10 ml after negative aspiration.

The procedure was started by the thoracic surgeon and team; biopsy was taken and sent for frozen section, which revealed squamous cell carcinoma. However, the nature (primary vs metastatic) was yet to be determined to guide lobectomy; therefore, further resection was not done, and port sites were closed after chest drain insertion. Intravenous paracetamol 1 g and ketorolac were given as part of multimodal analgesia along with dexamethasone and ondansetron 8 mg each for prevention of postoperative nausea and vomiting. The procedure lasted for one hour and 15 minutes. The patient was turned supine after completion of the procedure, and after confirmation of return of spontaneous breathing, muscle relaxation was reversed with neostigmine-glycopyrrolate 2.5/0.5 mg. The patient was successfully extubated after he started obeying commands, regained adequate muscle strength, and made adequate tidal volumes with regular respiratory rate thus fulfilling subjective and objective criteria for extubation. 

The patient complained of pain at the chest drain insertion site after waking up, which was managed with epidural bolus followed by infusion at 10 ml per hour. He was shifted to the PACU on five liters of supplemental oxygen via a tracheostomy mask in hemodynamically stable condition. He was discharged on the second postoperative day after removal of the epidural catheter and drains without any complication.

## Discussion

According to IARC 2022, laryngeal cancer is the 20th most common cancer worldwide with the 18th highest mortality rate [6]. According to WHO estimates, more than 180,000 cases are reported annually, and more than 50% end in death. The most common attributable risk factors are smoking and alcohol consumption [7]. Despite advances in treatment modalities, total laryngectomy remains the cornerstone of treatment. Those who survive have a significant risk of developing secondary primary malignancies of the head and neck, respiratory, or digestive tract [8]. It means more of the survivors will come for surgery in the coming times, and their airway management will pose unique challenges to the anesthesiologists.

Airway management in general and OLV in specific has its own set of challenges in post-laryngectomy patients. The size of tracheostoma has to be kept in mind during preoperative assessment and planning the intraoperative modality of airway management.

A number of ways have been used by anesthesiologists and described in the literature for post-laryngectomy patients requiring OLV for thoracic procedures, e.g., using DLT through tracheostoma, intubating the mainstem bronchus with a single lumen tube, use of bronchial blockers through single lumen tubes.

 In recent years, numerous case reports, small series, and reviews have been published focusing on OLV in tracheostomy or laryngectomy patients. This review summarizes the common techniques, available devices, clinical outcomes, and recommendations from the past years, and highlights knowledge gaps for future research.

Challenges in OLV for patients with tracheostomy or laryngectomy

Patients with tracheostomies or laryngectomies present several challenges for lung isolation. Key issues include the following:

Shortened Airway With Modified Anatomy

The distance from the tracheal stoma to the carina is much shorter than the distance from the mouth to the carina, leaving little length to accommodate standard devices. The tracheostomy site also creates an atypical angle that can make insertion and positioning of a rigid DLT difficult. An altered curvature of the airway (especially if a tracheostomy tube’s angle is fixed) can hinder the maneuvering of bronchial blockers or tubes [9,10].

Recent vs. Mature Stomas

If the tracheostomy is fresh (within <1 week), the tract is not well-formed and is prone to false passages or collapse if the tube is removed. Changing or removing a new tracheostomy tube risks losing the airway. In contrast, a well-established stoma (>7 days old) is less likely to close or false-track, allowing more flexibility in exchanging devices [1].

Upper Airway Patency

It is important to know why the tracheostomy was performed. If the upper airway is still patent and unobstructed, one option is to temporarily remove or occlude the tracheostomy and perform OLV with standard oral intubation techniques (though this may not be preferred if the trach was placed for an upper airway obstruction). In laryngectomy patients, the upper airway is disconnected entirely, meaning the neck stoma is the only route to the lungs [11].

Previous Radiotherapy and Surgery

Many laryngectomy patients have a history of head-neck cancer and may have received radiation. Fibrosis from radiation or surgery can distort airway anatomy and reduce tissue elasticity. Such patients might have tracheal stenosis at the stoma or fibrosis that complicates tube placement and increases the risk of airway tears [10,12].

Phonation Devices

Post-laryngectomy patients may have a tracheoesophageal voice prosthesis. This device sits in a fistula between the trachea and esophagus and can be displaced during intubation or manipulation. Careful preoperative assessment of the stoma size and the presence of any prosthesis is required, and a plan should be in place to prevent or manage inadvertent prosthesis displacement [13]. Some practitioners elect to remove the voice prosthesis temporarily during surgery to avoid losing it in the airway.

Airway Protection vs. Lung Isolation

The anesthesiologist must balance maintaining a secure airway with achieving lung separation [10]. These patients often have a difficult airway status to begin with. Ensuring oxygenation and ventilation are paramount, so any OLV approach must guarantee that a patent airway is never lost during device exchanges or manipulations.

Given these challenges, a meticulous plan is needed for OLV in tracheostomized patients. Preoperative evaluation should include inspecting the stoma (or performing bronchoscopy through it) to gauge diameter and any airway abnormalities [9,11]. Difficult-airway equipment (fiberoptic bronchoscopes, alternative airway devices) and backup plans (for ventilation or conversion to two-lung ventilation) should be prepared in advance.

Practices and devices for OLV in altered airway anatomy

Various techniques have been reported to achieve OLV in adult patients with tracheostomies or laryngectomies. The choice of technique depends on factors like the age of the stoma, its size/shape, and the equipment available [14]. Common OLV strategies in altered airways include:

Double-Lumen Tubes

Standard oral DLTs are often unsuitable in the presence of a neck stoma because of their length and size. A conventional DLT placed via a tracheostomy can easily be malpositioned - the shortened airway may cause the tube to advance too far, or the bulky tube may injure the stoma site. If a DLT is attempted, it usually needs to be a smaller size than normal to fit the stoma, and even then, it must be placed under bronchoscopic guidance to ensure correct positioning in the bronchus. There are case reports of DLTs being successfully used through tracheostomy stomas, but only with careful placement and confirmation by fiberoptic bronchoscopy [5,15]. To address these issues, specialized DLTs have been designed for tracheostomized patients. Notably, the Rüsch TracheoPart® DLT is a short DLT specifically made to be inserted via a tracheostomy stoma. It comes in multiple lengths (e.g., 75, 85, 95 mm) to accommodate the distance from stoma to carina, and has a curvature matched to sit securely at the stoma site. Such a device can provide true double-lumen functionality (independent lung ventilation, suctioning, etc.) while minimizing the risk of migration in a short trachea. If the patient’s upper airway is intact and the tracheostomy is not mandatory for breathing, another approach is to remove the tracheostomy tube and perform oral intubation with a DLT, then re-establish the trach later. This was suggested as an option in scenarios where the tracheostomy was done for lung pathology rather than upper airway obstruction [16]. In practice, however, DLT use in altered airways is less common due to the technical difficulties and risk of trauma [17].

Bronchial Blockers

BBs are frequently the preferred method of lung isolation in tracheostomy patients [1,9,14,18]. A BB is a catheter with an inflatable cuff that can be steered or positioned into one main bronchus to occlude it. In a tracheostomized patient, a BB can be placed through the existing tracheostomy tube or through a single-lumen endotracheal tube inserted via the stoma. This obviates the need to exchange the airway device. Numerous blockers have been used in this context, including the Arndt wire-guided blocker and Cohen flex-tip blocker (both Cook Medical), the Fuji Uniblocker, the Rüsch EZ-Blocker (a Y-shaped dual blocker), Fogarty arterial embolectomy catheters, and even modified Foley catheters as makeshift blockers [10]. For example, one recent case report describes using a 7.5 mm cuffed tracheostomy tube with a Coopdech bronchial blocker passed through it to achieve left lung isolation in a patient with a laryngectomy stoma [17]. The blocker was positioned into the left mainstem under fiberoptic guidance, successfully collapsing the left lung for surgery. The major advantage of bronchial blockers is that they can be deployed without removing a secured airway tube - this maintains continuous airway control and avoids needing to swap out an endotracheal tube at the end of the case [10]. Blockers also allow targeted blockade of a specific lobe if needed, and they tend to cause less trauma at the stoma since they are smaller in diameter than a DLT. Disadvantages include a slower lung collapse (since the lung deflates passively) and the inability to suction the isolated lung; any secretions or blood in the non-ventilated lung cannot be easily cleared. Moreover, blockers can be tricky to position through a trach - the curvature of a tracheostomy tube or the limited space alongside an existing tube may make it challenging to advance the blocker into the desired bronchus. There is also a tendency for the blocker to dislodge with patient movement or surgical manipulation, given the short and rigid path from stoma to bronchus. Frequent bronchoscopic checks or securing of the blocker’s position might be necessary. In summary, BBs are highly useful in these patients, and a recent six-year retrospective analysis found that using a bronchial blocker through a tracheostomy was safe and effective in the vast majority of cases [19]. Best practice is to use fiberoptic bronchoscopy to guide the blocker into position and confirm lung collapse before surgery. For very new tracheostomies (e.g., a fresh surgical airway), experts recommend against removing the trach or changing tubes; instead, one should use an intraluminal blocker through the existing tracheostomy tube to achieve OLV, thereby preserving the tract’s integrity [9,20].

Single-Lumen Endobronchial Intubation

In scenarios where a DLT will not fit and a blocker is unavailable or fails, a straightforward solution is intentional mainstem intubation with an SLT [21]. This involves advancing a cuffed endotracheal tube into one main bronchus to isolate the opposite lung. Several recent case reports describe successful OLV using this technique in laryngectomy patients. For instance, Imai et al. (2024) reported a case of a post-laryngectomy patient (with a tracheoesophageal voice prosthesis in situ) in whom a long, spiral-reinforced single-lumen tube (6.0 mm internal diameter) was inserted via the tracheostoma and navigated into the left main bronchus under bronchoscopic guidance [13]. This achieved OLV without disturbing the voice prosthesis, and the authors noted it was chosen due to the narrowed stoma that could not accommodate a DLT. In another case, a patient with a permanent tracheostomy after laryngectomy required left lung collapse for thoracotomy; anesthesiologists passed a 6.0 mm endotracheal tube into the right mainstem bronchus and separately insufflated oxygen to the left lung via a small-bore catheter to improve oxygenation [22]. Mainstem intubation via the stoma is technically simple and uses standard equipment, which can be an advantage in low-resource settings [22]. This technique guarantees a secured airway (the tube in the bronchus) but has notable drawbacks. If the right main bronchus is intubated, the right upper lobe will be unventilated (since the RUL bronchus departs before the right main bronchus entry of most tubes), leading to incomplete isolation and potential hypoxemia or difficulty achieving full collapse of that lobe [10]. OLV has also been achieved with MLT in a tracheostomized patient [23]. With a SLT, you also lose the ability to selectively ventilate or apply suction/CPAP to the non-ventilated lung on demand. Re-inflation of the collapsed lung for recruitment or testing can only be done by partially withdrawing the tube to the trachea, which is cumbersome. There are also safety concerns: overinflating the cuff in a mainstem could cause mucosal injury, and the tube can obstruct the bronchus if advanced too far. Because of these issues, single-lumen bronchial intubation is often considered a secondary option when other methods are unsuitable. If used, some of its limitations can be mitigated by techniques such as placing a suction catheter or thin lumen to the opposite lung to insufflate oxygen or provide CPAP (to avoid severe desaturation) [22]. All such cases demand vigilant monitoring - a bronchoscope should be used to ensure the ETT is correctly positioned in the intended bronchus, and the anesthesia team should be prepared to adjust if oxygenation or isolation is not adequate.

Other Adjunct Techniques

A few innovative approaches have been described in the literature. One example is using a cuffed tracheostomy tube as the OLV conduit: if the patient has a large-bore tracheostomy tube (for instance, a silicone adjustable-flange tracheostomy tube), the anesthesiologist can leave it in place and pass a bronchial blocker through it to the desired bronchus. Garg et al. reported a successful OLV using an adult-size adjustable flange tracheostomy tube combined with a blocker, which provided a secure and flexible airway in a laryngectomy patient with a history of radiation [24]. Another creative method is the extraluminal blocker technique using an LMA: in one report, an LMA was inserted orally (above the laryngectomy stoma, in a patient who still had a connection to the lower airway), and a bronchial blocker was passed through the LMA down into the trachea, then out through the tracheostomy, essentially using the LMA as a conduit to guide the blocker into the distal airway from above [25]. This allowed placement of a blocker without going through the angulated tracheostomy tube. Such techniques are highly specialized and would only be considered if conventional approaches fail [26]. Finally, regardless of the device, it is generally recommended to use a flexible fiberoptic bronchoscope during these cases: it aids in guiding tubes/blockers into the correct bronchus and is crucial for verifying lung isolation (correct placement is usually confirmed by direct visualization and by differential breath sounds [11].

Reported outcomes, complications, and procedural challenges

Published case reports and series in recent years indicate that with a tailored approach, OLV can be successfully achieved in tracheostomized or laryngectomized patients. Most reports describe favorable surgical and anesthetic outcomes; lung isolation was sufficient for the surgeons, and adequate oxygenation and ventilation were maintained throughout the procedure [11]. However, these cases also highlight several important complications and challenges.

Device Malposition, Malfunction, and Dislodgement

Because of the short airway and surgical manipulations, bronchial blockers in particular are prone to moving from their intended position. Even a slight shift can end ventilation of the operative lung or allow leak to the collapsed lung. Multiple authors note the need for frequent fiberoptic checks or adjustments of blockers during surgery [10]. DLTs inserted via a tracheostomy may also migrate; an undersized DLT might slide into one bronchus or be displaced with patient repositioning [11]. Securing the tube or blocker and minimizing circuit tension is critical. Two of the case reports showed fracture of the Coopdech bronchial blocker following insertion, successfully replaced with another one without airway compromise [27].

Inadequate Lung Collapse or Ventilation

Achieving complete lung collapse on the surgical side can be more difficult in these patients [18]. For example, if a right mainstem intubation is used for OLV, the right upper lobe is not isolated and may remain partially ventilated - this can reduce surgical exposure. In one case, this was managed by clamping the right upper lobe bronchus via bronchoscope, but that is not always feasible. Conversely, a blocker that occludes a bronchus but doesn’t allow active suction means the lung may take longer to deflate, potentially hindering the surgical view initially. Anesthetic teams have employed strategies like apneic oxygenation or low-pressure CO₂ insufflation to help collapse the lung faster, or applied a few centimeters of CPAP to the non-ventilated lung (through a small catheter) to improve oxygenation without re-expanding it [22]. Each approach must be individualized to the patient’s physiology and the surgical needs.

Hypoxemia and Ventilation Issues

OLV inherently carries the risk of hypoxemia due to shunt, and this risk can be higher if lung isolation is imperfect or if the patient has underlying lung disease. In tracheostomy/laryngectomy patients, OLV has been maintained successfully in most reported cases, but careful attention was required for oxygenation. The use of 5-6 cm H₂O CPAP to the collapsed lung via a thin catheter (as done in at least one report) can markedly improve oxygenation during OLV [22]. If oxygenation becomes unsatisfactory, the team should be ready to temporarily resume two-lung ventilation or adjust the blocker/tube position.

Airway Trauma

Manipulating equipment through a tracheostomy stoma can cause trauma. Large DLTs can stretch or tear the stoma site, leading to bleeding [17]. Even BBs, if repeatedly moved or if the bronchoscope and blocker crowd the tracheostomy tube, can cause mucosal injury [10]. The literature emphasizes using the smallest necessary devices and lots of lubrication to minimize trauma. Stoma injury is of particular concern in irradiated patients, where tissue healing is poor. In reported cases, significant bleeding or airway rupture has not occurred, but minor bleeding at the stoma or in the bronchus was occasionally noted and managed with suction and observation.

Loss of Airway Control

Any time a tracheostomy tube is exchanged or removed, there is a risk of losing the airway - either by the stoma closing or by the new tube failing to enter the trachea (false passage). This is why recent guidelines suggest not removing a fresh trach for lung isolation [11,17]. In the cases reviewed, most anesthesiologists kept a secure airway at all times (for example, only inserting a DLT after the patient was already intubated with a single lumen as a placeholder, etc.). No report described a catastrophic loss of airway, but it remains a critical hazard. Having rigid bronchoscopy or emergency cricothyrotomy kits on standby is prudent in these cases.

Special Situational Complications

In laryngectomy patients with voice prostheses, one notable risk is the prosthesis dislodging into the airway. The case by Imai et al. specifically planned around this; they opted for a smaller tube to avoid knocking the prosthesis out [13]. It’s advisable to either remove the prosthesis temporarily or ensure it is firmly secured and located (some teams suture a tether to it) before instrumentation. Another scenario is combined head-neck and thoracic procedures: sometimes an ENT surgeon can assist in managing the stoma or even perform a temporary occlusion of the tracheoesophageal puncture if needed during anesthesia.

Overall, the outcomes reported in recent literature are positive - OLV was achieved, and surgery proceeded successfully in these challenging patients. The key to avoiding complications is planning and vigilance. Nearly all authors stress the importance of continuous monitoring (with auscultation and bronchoscopy) and being prepared to adjust the plan intraoperatively. Close communication with the surgical team is also vital, so that any issues with lung isolation can be addressed together; for example, the surgeon might accept intermittent two-lung ventilation if needed or help reposition a blocker. 

Guidelines and expert recommendations

There are no formal society guidelines dedicated to OLV in tracheostomy or laryngectomy patients, largely due to the rarity of these cases. However, several expert recommendations and consensus points emerge from the recent literature:

Preoperative Planning

Thorough assessment of the airway is mandatory. This may include examining the stoma for size and stability, bronchoscopic evaluation of the trachea/bronchi for any stenosis or altered anatomy, and reviewing any prior tracheostomy or laryngectomy surgical details. If a voice prosthesis or stent is present, plan whether it will be removed or left in place (and secured). Imaging (chest CT) can be helpful to measure the distance from stoma to carina and the diameters of main bronchi, which inform the choice of tube size. Have a clear plan A, B, and C for airway management and lung isolation, and discuss these with the surgical team. For example, plan A: use bronchial blocker through trach; plan B: if unsuccessful, advance single-lumen into bronchus; plan C: occlude one lung surgically or ventilate both if isolation fails, etc. [10]. 

Choice of Technique Based on Stoma Age

A commonly cited approach is to use the “seven-day rule.” If the tracheostomy is recent (<7 days), avoid removing or changing the tracheostomy tube if at all possible [1]. Instead, perform lung isolation through the existing tube (typically with a bronchial blocker) to prevent disrupting the fresh tract. If the tracheostomy is older (>7 days) and the stoma is mature, one has more options: the tracheostomy tube can be safely exchanged for a specialized DLT or a single-lumen ETT if needed [17]. This guideline is supported by clinical experience that recent stomas can close or false-track easily, whereas mature stomas behave more like a fixed airway opening.

Upper Airway Utilization

Determine if the upper airway can be used for OLV. If the patient has an intact larynx and the tracheostomy was done for, say, prolonged ventilation (not for obstruction), some experts recommend intubating orally with a DLT or a SLT and either closing the trach stoma or intubating it with a second tube for ventilation as needed [17]. In practice, most cases in the literature with permanent stomas (like laryngectomies) had to use the stoma, but in tracheostomized patients with a partially functional upper airway, it’s worth considering the conventional route. Any plan to use the upper airway must account for securing the trach site (to avoid air leak) and be ready to revert to the stoma if intubation fails.

Fiberoptic Guidance

All recent reports and reviews emphasize that lung isolation in these patients should be performed with bronchoscopic guidance. Whether placing a DLT or a blocker, or a single-lumen, a flexible bronchoscope greatly increases the accuracy of placement and reduces complications. It allows direct visualization of the blocker or tube entering the target bronchus and can confirm that the other lung is indeed excluded (e.g., seeing the blocker’s cuff occluding the bronchus, or seeing the DLT’s bronchial lumen in the correct bronchus). Fiberoptic bronchoscopy is also invaluable for troubleshooting during OLV - if there’s sudden loss of isolation or ventilation, one can quickly inspect for blocker migration or secretion plugging. Therefore, the recommendation is to have a fiberoptic scope immediately available and to use it proactively during placement and periodically during the case [2,5].

Equipment Selection

Have a range of airway devices on hand. This includes different sizes of endotracheal tubes (one should have small-diameter, long tubes available - e.g., reinforced tubes 6.0-7.0 mm for possible bronchial intubation in a narrow stoma) [16]. If possible, obtain a bronchial blocker kit (Arndt or Cohen blocker, etc.) and ensure your team knows how to use it. If a specialized tracheostomy DLT (like the Rüsch TracheoPart) is available in your setup, it can be an excellent choice for appropriate patients. However, these devices may not be available in all hospitals. In their absence, a small conventional DLT (e.g., 26 Fr or 28 Fr for adults) might be used as a substitute, but again only in mature stomas and with caution [18]. Also consider having suction catheters that can be passed alongside an ETT if needed to provide oxygen insufflation or CPAP to the collapsed lung, as this can be a simple way to prevent desaturation [19].

Intraoperative Management

Once lung isolation is achieved, secure everything. For a tracheostomy tube or DLT at a stoma, anchoring the tube well (with ties around the neck and perhaps a suture) can prevent accidental decannulation or movement. The patient’s position (lateral decubitus for thoracic surgery) can pose an additional risk of tube displacement, so all circuits and scopes should be carefully positioned to avoid torque on the airway. Continually monitor bilateral breath sounds and oxygenation. Many practitioners keep the bronchoscope connected to the monitor for intermittent checks of blocker position. If surgical conditions are suboptimal (e.g., lung not fully collapsed), communicate with the surgeons - a small tweak like adding suction to the blocker lumen or transiently clamping the tracheostomy tube can help the lung deflate faster. Also, be prepared for the end of surgery: if a blocker was used, you can simply deflate and remove it, continuing ventilation through the tracheostomy tube. If a DLT was used via the stoma, you will likely need to exchange it for a single-lumen tracheostomy tube for postoperative ventilation - plan to do this with a tube exchanger or fiberoptic if the patient is still intubated deeply at the end.

Postoperative Care

After OLV in a patient with an altered airway, ensure the airway is stable for transport. If a new tracheostomy tube was inserted (or reinserted), verify its position and security. Watch for any airway edema or bleeding at the stoma site. Extubation or decannulation plans should take into account why the trach existed; many of these patients will remain with a trach post-op (e.g., laryngectomy stoma is permanent). Multidisciplinary involvement (thoracic surgeons, ENT surgeons, anesthesiologists) is beneficial in managing the airway in the immediate postoperative period if any issues arise.

In summary, the expert consensus is that there is no single best technique for all altered-airway patients; instead, anesthesiologists should be familiar with multiple lung isolation tools and choose the approach that fits the patient’s anatomy and clinical situation. Bronchial blockers are often the first-line choice due to their versatility and safety profile in a difficult airway. DLTs can be used in select cases, especially if suctioning or faster collapse is needed, but one must weigh the risks. Whichever method is chosen, careful planning, proper equipment, and skilled use of the fiberoptic bronchoscope are the cornerstones of success.

Gaps in the literature and future directions

Despite the growing number of case reports, high-quality evidence on OLV in tracheostomy or laryngectomy patients remains limited. There are no randomized trials or large prospective studies, understandably, since these clinical scenarios are uncommon and heterogeneous. Most recommendations are based on expert opinion or anecdotal experience. A lack of standardized guidelines has been noted in the literature journals, indicating a need for more research and consensus-building in this area. Some gaps and potential future research directions include:

Comparative Outcomes of Techniques

It is still unclear if one approach (e.g., bronchial blocker vs DLT vs mainstem intubation) is superior in terms of patient outcomes or safety. Future studies could compile multicenter experiences to compare success rates and complication rates of different OLV techniques in altered airways. Even a large registry or case series could help identify which methods are most reliable in practice.

Device Innovation

The development of specialized devices like the TracheoPart® DLT is a step in the right direction, but such tools are not widely available. Further innovation is encouraged - for example, creating adjustable-length DLTs or dedicated bronchial blockers designed for tracheostomy use (with steerable tips that account for the stoma angle). Simulation studies or cadaver labs could be used to test new device designs for tracheostomy OLV before they are introduced clinically.

Protocol and Training Development

Anesthesia providers would benefit from simulation-based training on how to manage OLV in a patient with a trach or laryngectomy. Developing simulation scenarios or difficult airway course modules for this situation could improve practitioner readiness, given how high-stakes and time-sensitive these cases can be. Additionally, formalizing an algorithm (much like the difficult airway algorithm) for OLV in tracheostomy patients could be useful. Campos et al. previously suggested an algorithm based on tracheostomy age and available equipment; building on that and incorporating recent device options could yield a consensus guideline that institutions adopt [1].

Adjunct Technique Research

More data on supportive measures, like the use of CPAP to the non-ventilated lung or high-flow apneic oxygenation during OLV, would be helpful. For instance, studying how 5 cm H₂O CPAP via a catheter affects oxygenation and surgical conditions in these patients could validate its routine use. Similarly, research into optimal fiberoptic techniques (such as whether to leave a bronchoscope in place as a blocker guide or not) may refine the procedure.

Managing Complex Airway Pathology

Some tracheostomized patients have additional challenges like tracheal stenosis, distal tumors, or severe tracheomalacia. Reports focused on these subgroups (e.g., OLV in a patient with tracheostomy and distal tracheal stenosis) are sparse. Future case studies or series that address these could fill important gaps.

Long-Term Outcomes

It could be valuable to track if OLV in a tracheostomy patient leads to any long-term stoma complications (does inserting a DLT via stoma cause any later tracheal scarring or enlargement? Does blocker use have any lasting impact?). So far, the literature hasn’t reported on longer-term follow-up of the airway itself after these interventions.

Finally, as more experiences are published, professional societies (such as anesthesia and thoracic surgery groups) may eventually incorporate guidance for altered airway lung isolation into their practice guidelines. This would help disseminate knowledge to all practitioners. Until then, clinicians must rely on careful interpretation of available case reports and reviews - like those discussed above - to inform their management. The encouraging news is that, with proper planning and technique, even these difficult cases can achieve successful OLV and favorable surgical outcomes, as evidenced by numerous recent reports. Continued sharing of experiences and incremental research will further improve safety and efficacy in this challenging aspect of anesthetic practice.

**Table 1:** Literature review of case reports, case series, and reviews on one-lung ventilation in tracheostomy/laryngectomy patients.

Author(s),Year	Study Design	Sample Size	Procedure	OLV Technique	Bronchoscopy Used	Outcomes Measured/Complication	Strengths	Limitation	Conclusion
Campos et al. (2018) [1]	Retrospective data analysis	70 patients out of 3,225 screened	Elective thoracic surgeries	Early-stage group (n=6): Shiley tube + bronchial blocker. Long-term group (n=64): SLT + blocker (38 cases), Shiley + blocker (15), SLT guided into a selective bronchus (7), DLT (4)	Used in all 70 cases	No complications occurred secondary to airway management or OLV.	Large sample size, focus on safety, clear categorization	Retrospective design, single-center experience	The authors recommend: Early tracheostomy (<7 days). Shiley tube + bronchial blocker. Long-term tracheostomy (≥7 days). SLT directly into one bronchus OR SLT/Shiley tube + bronchial blocker. DLTs are the least frequently used device
Ashok and Francis (2018) [2]	Educational review	-	-	1: Insert DLT through stoma after removing TT. 2: Insert SLT + BB through stoma after removing TT. 3: Pass BB through existing TT. 4: Replace TT with short DLT; 5: Orotracheal DLT or BB after TT removal (not in post-laryngectomy)	Recommended to confirm DLT or BB placement, especially in tracheostomy cases.	-	Specifically addresses OLV in tracheostomized patients	Does not include patient outcomes or clinical data.	This review establishes multiple safe OLV techniques for adult patients, including those with tracheostomies
Andros et al. (1993) [3]	Case report	1	Thoracoscopic pleurodesis via video-assisted thoracoscopy	OLV via tracheostomy stoma using a Univent endotracheal tube after tracheostomy tube removal.	Yes	The airway strategy was well tolerated, with no adverse events reported.	Highlights OLV in tracheostomy with tracheobronchial disease	No comparison with other techniques	The method is feasible, safe, and effective in complicated tracheostomy cases. Tracheobronchial narrowing precluded DLT use.
Shih et al. (2010) [5]	Case series	2	Case 1: Right pulmonary decortication. Case 2: Elective surgical tracheostomy followed by left pulmonary decortication	A size 28 DLT through the tracheostomy in both cases	Yes	Successful OLV via tracheostomy; no intraoperative complications.	Shows successful DLT placement via both permanent and fresh tracheostomies for OLV, cost-effective alternative to bronchial blockers	Small sample size; single-center experience	DLT via tracheostomy enables effective OLV with better cost-benefits than bronchial blockers.
Collins et al. (2018) [9]	Narrative review article	-		1: Bronchial blocker through the tracheostomy tube; 2: Small-sized double-lumen tube (DLT) via tracheostomy; 3: Oral placement of a DLT or bronchial blocker if anatomy allows; 4: Bronchial blocker through a cuffed tracheostomy tube	Recommended	-	Evidence-based recommendations, mentions tools like multiport adapters and specialized blockers	success rates, complication rates not discussed, Rüsch DLT unavailable in some regions, limiting its use.	For tracheostomy patients, fiberoptic-guided bronchial blockers are the safest and most effective method for lung isolation
Fernandes et al. 2024 [10]	Case report	1	Laparoscopic/thoracoscopic Ivor Lewis esophagectomy	Direct intubation of the left main bronchus with a single-lumen ETT (final technique)	yes	Right lung isolation, but 1: Required frequent repositioning, 2: Lung deflation was incomplete, 3: Suboptimal surgical field	Highlights lung isolation challenges and multiple techniques used in one tracheostomized patient case.	Single patient case	Tracheostomized patients pose airway challenges during OLV, often managed with BBs or DLTs.
Konduri et al. (2024) [11]	Narrative review	-	-	1: Bronchial blockers (intraluminal or extraluminal); 2: DLTs; 3: If feasible: Replace tracheostomy with oral DLT or ETT + BB	Recommended	-	Multiple techniques adaptable to early and late stoma	No outcome comparison	Selection depends on stoma timing and size: <7 days: Use BB via existing tracheostomy tube >7 days: Consider tube exchange for DLT or ETT + BB
Imai et al. (2023) [13]	Case report	1	Right lung segmentectomy	Long, cuffed, spiral single-lumen tube (SLT) through tracheostoma	Yes	Hypoxemia, SpO₂ dropped to 82% due to accidental tube migration into the left lower lobe	1: Provides algorithm f based on tracheostoma size and surgical procedure. 2: Details OLV approach in laryngectomized patients with VP in situ.	Single-case experience	Successful OLV was achieved using a long spiral SLT in a post-laryngectomy patient with a VP and narrowed tracheostoma.
Yamaguchi et al. (2024) [15]	Case report	1	Right lung lobe resection	Awake tracheostomy with subsequent exchange to DLT via tracheostomy site.	Yes	Temporary desaturation	1: Innovative use of a DLT through a tracheostomy site 2: Anticipated potential complications due to the laryngeal web and planned a preoperative tracheostomy instead of risking difficult oral intubation.	Single Case	Preoperative tracheostomy enabled safe one-lung ventilation using a DLT in a patient with laryngeal web.
Brodsky and Lemmens (2003) [17]	Prospective observational study	1170 (2 had tracheostomy)	Not mentioned	Case 1: DLT size 37 was inserted directly through the tracheostomy stoma. Case 2: Failed intubation led to tracheostomy; a 37F left DLT was inserted through the stoma	Not mentioned	Not mentioned	Involves 1,170 consecutive patients over eight years, include patients with tracheostomy	No procedural details	Left DLTs are safe for most thoracic surgeries. Size and depth should match airway anatomy. Confirm position; bronchoscopy is helpful but not always essential.
Ng et al. (2025) [14]	Case report	1	Left upper and lower lobe wedge resection with lymph node dissection	Laryngostomy tube exchanged for cuffed Portex tracheostomy tube; OLV achieved via intraluminal Coopdech BB to left main bronchus	Yes	None	Strong comparison and data tables, The presence of a mature laryngostomy allowed for straightforward tracheostomy tube exchange	No long-term or postoperative outcome discussed	This case shows safe, effective method for OLV in end tracheostomy, offering reliable lung isolation in altered airway anatomy.
Taghavi Gilani et al. (2015) [21]	Case report	1	Thoracic duct ligation	Dual ETT technique was used for OLV in a patient with tracheostomy stenosis. Two 4.5 mm tubes were placed into each bronchus via fiberoptic scope; the right tube was clamped for left-lung ventilation.	Yes				
Yousuf et al. (2023) [22]	Case report	1	Left open thoracotomy and lower lobe excision	Single-lumen ETT placed in right main bronchus; CPAP to non-ventilated lung via the Nelaton catheter	Yes	Successful OLV, no complications	Novel, low-cost technique,	Single patient case; no comparison with standard techniques	Low-trauma alternative for OLV in patients with permanent tracheostoma post-laryngectomy.
Howell et al. (2014) [23]	Case report	1	right thoracotomy and lung decortication	5.0 mm MLT was orally inserted into the left main bronchus as an EBT under fiberoptic guidance, with the tracheostomy tube left in place and both connected via a double-swivel adapter.	Yes	Successful OLV, no complication	Novel technique, reduces risk of airway loss from removing a fresh tracheostomy.	This technique demands expertise	An effective OLV method for recent percutaneous tracheostomy, using a microlaryngeal tube as a left endobronchial tube without removing the tracheostomy—suitable when BBs or DLTs are not.
Garg et al. (2016) [24]	Case report	1	Left upper lobectomy and mediastinal lymph node dissection.	OLV was achieved via tracheostomy using a silicone hyperflex adjustable flange tracheostomy tube (SHATT) with an Arndt endobronchial blocker in the left main bronchus	Yes	Successful OLV, no complication	SHATT offered flexibility and stability for altered anatomy.	Requires specialized equipment	SHATT with Arndt blocker enables safe OLV in tracheostomized patients
Venkataraju et al. (2010) [27]	Case report	2	Case 1: VATS lung biopsy. Case 2: VATS bullectomy and pleural abrasion	Coopdech bronchial blocker via a single-lumen endotracheal tube.	yes	Case 1: Fracture of the blocker tip, requiring extubation and reinsertion. Case 2: Tip deformation due to entrapment at the Murphy’s eye, requiring replacement.	Highlights real and rare complication,	No long-term follow-up	The Coopdech bronchial blocker’s angulated tip poses a risk of fracture during insertion, especially in right bronchial placements, necessitating careful handling and continuous bronchoscopic guidance.

## Conclusions

OLV in post-laryngectomy patients is challenging due to altered anatomy and limited airway management options, yet with careful planning and fiberoptic guidance, safe lung isolation can be achieved. Our case highlights that a DLT inserted via a mature tracheostoma is a feasible approach, while the literature supports alternative strategies such as bronchial blockers or mainstem intubation depending on patient factors and equipment availability. Across reports, three key principles consistently emerge: securing the airway before lung isolation, confirming placement with fiberoptic bronchoscopy, and maintaining backup strategies. Given that current evidence is largely case-based, individualized management and multidisciplinary collaboration remain essential until more standardized guidelines are developed.
